# Antibacterial Mechanism and Flavour Impact of Ultrasound and Plasma-Activated Water Combination on *Aeromonas veronii* in Crayfish

**DOI:** 10.3390/foods14060926

**Published:** 2025-03-08

**Authors:** Weicheng Xu, Rongxue Sun, Zhanke Qin, Ziai Deng, Yi Liu, Haojie Zhang, Haibo Luo, Ning Jiang, Hao Cheng, Maozhi Ren

**Affiliations:** 1 Institute of Urban Agriculture, Chinese Academy of Agricultural Sciences, Chengdu National Agricultural Science and Technology Center, Chengdu 610213, China; 2 Department of Agricultural Engineering, Kizilsu Vocational Technical College, Kizilsu 845350, China; 3 Institute of Agricultural Products Processing, Jiangsu Academy of Agricultural Sciences, Nanjing 210014, China; 4 School of Food and Pharmaceutical Engineering, Nanjing Normal University, Nanjing 210023, China

**Keywords:** *A. veronii*, food microbiology, sterilisation mechanism, disinfection, spoilage organisms, food safety

## Abstract

*Aeromonas veronii* is a foodborne pathogen commonly found in contaminated crayfish. In this study, the effects of ultrasound combined with plasma-activated water (US-PAW) against *A. veronii* and on the flavour of crayfish were investigated to evaluate their impact on crayfish preservation. In vitro, US and PAW showed a significantly synergistic inhibition against *A. veronii* growth and biofilm reformation at 7 min. Furthermore, PAW disrupted the membrane integrity of *A. veronii*, accompanied by enhanced outer membrane permeability, with bacteria exhibiting distortion, deformation, and the accelerated leakage of intracellular substances, which US-PAW further promoted. Additionally, US-PAW increased the intracellular levels of reactive oxygen species and hydrogen peroxide, disrupting cellular homeostasis. This resulted in a significant decrease in the activities of SOD and GSH, as well as a reduction in the intracellular ATP concentration and the activities of MDH and SDH. The results indicated that US-PAW treatment impairs the ability of *A. veronii* cells to generate sufficient energy to resist external stress, ultimately leading to bacterial death due to the inability to maintain normal physiological functions. According to the bacterial cell count and GC-MS analysed, US-PAW treatment increased the storage period of crayfish (infected with *A. veronii*) by 2 days, while reducing sulphur-containing volatiles within 24.64% during 6 days of storage at 4 °C. These conclusions provide a theoretical foundation for the industrial application of US-PAW to preserve crayfish.

## 1. Introduction

The consumption of aquatic products is an indispensable component of the global consumer diet. Crayfish are highly regarded for their nutritional value, distinctive flavour, tender texture, and vibrant colour. However, crayfish, fish, molluscs, and other aquatic products are prone to microbial contamination during processing, transportation, and storage [1]. This could result in a rapid deterioration in quality, shortened shelf life, and reduced economic value. *Aeromonas* is a common Gram-negative, rod-shaped spoilage bacterium (also human pathogenic bacterium) in crayfish, and it is widely distributed in freshwater environments [2,3]. The typical symptom of *Aeromonas* infection in humans is haemorrhagic septicaemia. Reports indicate that the incidence of haemorrhagic septicaemia caused by *Aeromonas* in France, the United Kingdom, and Southern Taiwan is 0.66, 1.5, and 76 cases per million individuals, respectively [4]. Therefore, foodborne diseases caused by *Aeromonas* have become a public health concern shared by many countries. As a result, there is an urgent need to develop new technologies that enhance the microbial safety and quality of crayfish-related foods in a safe, effective, and environmentally sustainable manner.

Currently, physical methods are widely employed for the disinfection and decontamination of crayfish in many countries. Traditional physical technologies such as heat treatment, ultra-high pressures, and high-power ultrasound (US) are effective in inhibiting microbial activity and extending the shelf life of crayfish. However, they lead to compound degradation or oxidation and potentially impact the food matrix’s stability, while being associated with a high energy consumption and operational costs [5]. Compared to traditional physical disinfection methods, US combined with other techniques could significantly enhance sterilisation efficiency, reduce the processing time and intensity, preserve flavour characteristics, and promote energy conservation [6]. For example, Sagong et al. applied US combined with 2.0% malic acid for the surface sterilisation of lettuce and found that at a lower US power (90 W) and shorter treatment time (5 min), the *Salmonella* population in lettuce was reduced by 2.73 log CFU/g [7]. Similarly, Park et al. used US in conjunction with mildly acidic electrolysed water and observed a significant reduction in the number of *Vibrio parahaemolyticus* and *Escherichia coli* in herring, with *V. parahaemolyticus* decreasing by 1.42 log CFU/g and *E. coli* by 1.86 log CFU/g [8]. Consequently, the innovation of combining US with other techniques represents a key development in the field of food disinfection and decontamination.

Plasma-activated water (PAW) is a unique biochemical reaction chemical mixture (HNO_3_, OH, HNO_2_, ONOO^−^, O_2_^−^, O_3_, H_2_O_2_) produced by the plasma treatment of water and triggering several chemical reactions [9]. The active species in PAW degrade rapidly over time. However, the formation and collapse of US-induced bubbles can reach critical temperature and pressure conditions, causing the acoustic dissociation of the water and the generation of free radicals (such as -H and -OH), which can reactivate the residual free radicals in PAW [10]. In addition, the cavitation effect of US increases the contact area between PAW and food, maximising microorganism reduction [11]. Therefore, ultrasound combined with plasma-activated water (US-PAW) has recently been widely recognised as a new, efficient, sustainable disinfection technology. Due to its antimicrobial activity, US-PAW has been widely applied in food preservation for fruits, vegetables, meat, baked goods, and aquatic products [12,13,14]. Moreover, the addition of US-PAW did not generate adverse effects on the organoleptic properties of the crayfish [15].

Despite these advancements, a critical gap in our knowledge exists concerning the specific antimicrobial mechanisms and flavour effects of US-PAW on crayfish spoilage bacteria. The widely recognised representative of *Aeromonas* in freshwater environments is *Aeromonas veronii* (*A. veronii*), identified in previous studies as the dominant spoilage bacterium in crayfish [16]. To shed light on this issue, the present study focuses on US-PAW. It compares it with US or PAW alone in terms of the morphology, cell membrane integrity and permeability, antioxidant enzyme systems, and energy metabolism systems of *A. veronii* under optimal conditions. Finally, the effect of US-PAW on the antibacterial activity of *A. veronii* in crayfish, along with the associated changes in crayfish flavour, is analysed.

## 2. Materials and Methods

### 2.1. Sample Preparations

#### 2.1.1. Bacterial Strain and Working Solution Preparations

In previous investigations, the *A. veronii* strain was isolated and purified as a predominant spoilage bacterium from deteriorated crayfish [16]. The strain of *A. veronii* was incubated in sterile tryptone soy broth (TSB, Hopebio Technology Co., Ltd., Qingdao, China) at 37 °C, shaken at 180 r min^–1^ for 24 h. Subsequently, the culture was spread onto plate count agar (PCA, Hopebio Technology Co., Ltd., Qingdao, China) to isolate individual colonies. To generate a working solution with a final concentration of 10^8^ CFU/mL, a separate colony was selected and propagated TSB under identical incubation conditions as previously described.

#### 2.1.2. Crayfish Collection and Preparation

Fresh crayfish (*Procambarus clarkii*) were sourced from Huai’an Fishermen Aquatic Products Co., Ltd., Huaian, China. For experimental samples, individuals matching closely in weight (25 ± 2 g), size (8 ± 1 cm), and superficial colouration (red shell) were selected. The crayfish were encased in sealed polyethene containers with a ratio of crayfish to ice of 1:2 (*w*/*w*) and transported to the laboratory within 1 h. Upon arrival, the crayfish were immediately subjected to death by immersion in ice water, promptly followed by the ensuing procedures.

### 2.2. US and PAW Preparations

For the US treatment, a 4 L ultrasonic tank (model KQ-100DE, Ultrasonic Instrument Co., Ltd., Kunshan, China) was utilised, filled with 3 L of distilled water. The operational parameters of the ultrasonic device were established as a frequency of 40 kHz and a power setting of 100 W. Throughout the experiment, the temperature was meticulously maintained below 25 °C.

In this investigation, PAW was generated using a non-thermal atmospheric pressure plasma system (PG-1000Z/D, Nanjing Suman Electronics Co., Ltd., Nanjing, China); for a detailed description of the equipment, readers are referred to previous studies [11].

### 2.3. Optimisation of Preparation Conditions for US-PAW

In this work, the parameters of the PAW activation time, US treatment time, and water volume for the preparation of US-PAW were optimised by measuring the physicochemical properties of US-PAW. Based on the characteristics of the PAW activation time (0, 1, 3, 5, 7 and 10 min), the US treatment time (0, 5, 10, 15, 20, and 25 min) and water volume (200, 400, 600, 800, 1000, and 1500 mL) were tested in this study. The oxidation–reduction potential (ORP) and pH value of the US-PAW were immediately determined after the US-PAW’s generation using a Leici multimeter (DZS-708 T, Lei Ci Co., Ltd., Shang Hai, China). The concentration of hydrogen peroxide (H_2_O_2_) in the US-PAW was measured by a hydrogen peroxide test kit (S0038, Beyotime Institute of Biotechnology Co., Ltd., Shanghai, China). The concentration of hydrogen peroxide was determined based on the oxidation of ferrous ions (Fe^2^^−^) to ferric ions (Fe^3^^−^) by hydrogen peroxide, followed by the formation of a purple-coloured complex through the reaction between the generated Fe^3^^−^ and xylenol orange in a specific acidic medium. This chromogenic reaction enabled a quantitative analysis through spectrophotometric measurement at the A560 nm wavelength of the resultant complex. The concentrations of ozone (O_3_), nitrite (NO_2_^−^), and nitrate (NO_3_^−^) were analysed by a UV–Vis spectrophotometer (UV-6300, Mapada Instruments Co., Ltd., Shanghai, China) as described previously [17,18].

### 2.4. Sample Treatments

#### 2.4.1. Bacterial Treatment

In brief, 1 mL of working solution of *A. veronii* was centrifuged at 8000× *g*, 4 °C for 5 min. The supernatant was discarded, and the bacterial pellet was washed three times with PBS (0.01 M, pH 7.2). The bacterial pellet was resuspended in 10 mL sterile water or PAW as the CK (untreated) or PAW group. For the US or US-PAW group, 10 mL of sterile water or PAW was added to the bacterial pellet, followed by US treatment in a water bath. At defined time points (1, 3, 5, 7, 9, 10, 12, 14, and 15 min), the samples were 10-fold serially diluted with PBS. A 100 μL aliquot of each dilution was plated on PCA plates and incubated at 37 °C for 24 h, after which the colonies were counted.

(1) $$N=\frac{\sum C}{\left(n_{1}+0.1n_{2}\right)d}$$

*N* is the bacterial cell count (log CFU/mL), ∑C is the sum of the number of colonies on plates (plates with an appropriate range of colony counts), *n_1_* is the number of plates at first dilution (low dilution), *n_2_* is the number of plates at second dilution (high dilution), and *d* is the dilution factor (first dilution) [19].

(2) $$I=1-\frac{A_{0}}{A_{1}}\;$$

*I* is the inhibition rate, *A*_0_ is the bacterial cell count of the untreated group, and *A*_1_ is the bacterial cell count of the treatment group.

#### 2.4.2. Antibiofilm Formation

The antibiofilm activity of US-PAW was evaluated using a previously reported method [20]. Briefly, 2.5 mL of working suspension (approximately 10⁸ CFU/mL) was added to a 6-well microplate and incubated at 37 °C without shaking for 48 h. After incubation, planktonic cells were removed by inverting the microplate and washing it twice with ddH₂O. Then, 1 mL of sterile water or PAW was added to the biofilm and incubated as the CK or PAW group for the specified time. For the US or US-PAW groups, 1 mL of sterile water or PAW was added, followed by ultrasonic treatment for the specified time. The treated biofilm was washed with ddH_2_O dispersed into a bacterial suspension, and the second-generation biofilm was cultured under the same conditions. Biofilm biomass was quantified by staining 200 μL of 0.1% crystal violet (CV) at 37 °C for 20 min, followed by washing with ddH_2_O and ethanol elution. Absorbance at OD570 nm was measured using a standard microplate reader.

#### 2.4.3. Crayfish Inoculation and Treatment

To evaluate the effects of US, PAW, and their combination on *A. veronii* in crayfish, all the specimens underwent standardised microbial decontamination. Following the aseptic excision of their intestinal tracts to eliminate endogenous microbiota, the three crayfish samples were submerged in 75% ethanol for 1 h of disinfection and then exposed to UV light for 1 h. The three crayfish samples were then immersed in a 200 mL *A. veronii* bacterial suspension for 15 min, followed by air-drying for 1 h at room temperature (25 °C) in a biosafety cabinet to allow bacterial attachment. The bacterial load in the crayfish post-inoculation was 4.52 ± 0.13 log CFU/g. No colony growth was observed on plate count agar (PCA) plates from the crayfish samples without an inoculation. For the CK group, three crayfish samples (ca. 80 g) were immersed in a 1000 mL glass beaker containing 500 mL water, and were left to stand or periodically stirred for 40 min. For the PAW group, three crayfish samples were immersed in a 1000 mL glass beaker containing 500 mL PAW and treated for 40 min. For the US group, three crayfish samples were immersed in 500 mL sterile water in a 1000 mL glass beaker and subjected to ultrasonic treatment for 40 min. For the US-PAW group, three crayfish samples were immersed in 500 mL PAW in a 1000 mL glass beaker, placed in the centre of the ultrasonic bath, and treated with ultrasound for 10 min, followed by further stirring in PAW for 30 min. Untreated samples served as the control. At specified time points (0–8 days), the homogenate of crayfish samples was subjected to 10-fold serial dilutions with PBS. A 100 μL aliquot of each diluted suspension was spread onto PCA plates for the viable *A. veronii* counts, with three replicates per dilution. The plates were incubated at 37 °C for 24 h, after which plates containing 30–300 colonies were selected for counting.

### 2.5. Scanning Electron Microscopy (SEM)

Each bacterial pellet was fixed in 1 mL of pre-cooled 2.5% glutaraldehyde within a 2 mL centrifuge tube for 12 h, followed by dehydration through a gradient series of ethanol. Subsequently, 10 μL of each bacterial suspension was applied onto slides, dried using a supercritical dryer for one hour, coated with gold, and examined under scanning electron microscopy (SEM) with an EVO-LS10 device, Carl Zeiss Co., Ltd., Oberkochen, Germany.

### 2.6. Measurement of Cell Membrane Damage

#### 2.6.1. Measurement of Cell Membrane Integrity

A bacterial viability/virulence assay kit (L6060S, Uelandy Biotechnology Co., Ltd., Suzhou, China) was used to detect changes in cell membrane integrity. The bacterial viability/virulence assay kit contains two fluorescent dyes: NucGreen (green) labels all bacteria regardless of membrane integrity, while EthD-III (red) specifically stains membrane-damaged cells. Bacteria with intact membranes appear green, whereas those with compromised membranes exhibit both green and red signals under dual-channel detection, enabling the quantitative differentiation of viable and non-viable populations. The post-treated bacterial pellets were resuspended in 1 mL of sterile 8.5 g/L NaCl (Sinopharm). 10 μL of staining working solution (1 μL of NucGreen, 2 μL of EthD-III, and 7 μL of 0.85% NaCl (Sinopharm)) was added to 1 mL of each bacterial suspension, mixed well, and incubated for 15 min at room temperature in the dark. The examination was performed on a Confocal laser scanning microscope (CLSM) (Ultra View VOX, PE Co., Ltd., Wellesley, MA, USA).

#### 2.6.2. Electrical Conductivity

An *A. veronii* suspension (1 mL) was centrifuged (8000× *g*, 4 °C, 5 min), washed thrice with PBS (0.01 M, pH 7.2), and resuspended in 10 mL sterile water (CK group) or PAW (PAW group) for 7 min. For the US and US-PAW groups, the pellet was resuspended in 10 mL sterile water or PAW, respectively, and treated with ultrasound in a water bath for 7 min. The electrical conductivity of the post-treated bacterial suspensions was determined by a multimeter (DZS-708T, Lei Ci Co., Ltd., Shanghai, China).

#### 2.6.3. Leakage of Proteins and Nucleic Acids

Nucleic acid and protein leakage from *A. veronii* was quantified as described by Huang et al. [21]. After treatment, the bacterial suspensions were centrifuged at 8000× *g* for 5 min at 4 °C. Each sample’s supernatant (750 μL) was transferred to a quartz cell for absorbance measurement using a UV–Vis spectrophotometer (UV-6300, Mapada Instruments Co., Ltd., Shanghai, China).

#### 2.6.4. Permeability Analysis of the Outer Membrane

The outer membrane permeability was measured by the N-Phenyl-1-naphthylamine (NPN) fluorescent probe described by Helander et al. [22] with minor modifications. The post-treated bacterial pellets were resuspended in PBS (0.01 M, pH 7.2) and then added to acetone stock solution containing NPN (final concentration of 10 μmol/L), followed by incubation for 10 min at room temperature in darkness. The fluorescence intensity was measured using a fluorescence spectrophotometer (Cary Eclipse, Agilent Technologies Co., Ltd., Santa Clara, CA, USA).

### 2.7. Intracellular Reactive Oxygen Species (ROS) and H_2_O_2_ Content

Intracellular ROS levels in the bacteria were quantified using a ROS detection kit (R6033, Uelandy Biotechnology Co., Ltd., Suzhou, China). The non-fluorescent DCFH-DA permeates viable cells and undergoes intracellular esterase-mediated hydrolysis to membrane-impermeable DCFH, which is subsequently oxidised by ROS to fluorescent DCF. Fluorescence intensity quantitatively reflects dynamic changes in intracellular ROS levels. The DCFH-DA probe was diluted in PBS (0.01 M, pH 7.2) and added to the bacterial pellets to achieve a cell density of 10^7^ CFU/mL, followed by incubation in the dark at room temperature for 15 min. Fluorescence intensity was measured with a spectrophotometer (Cary Eclipse, Agilent Technologies, Santa Clara, CA, USA), and fluorescence imaging was performed using CLSM. The H₂O₂ concentration was determined with a hydrogen peroxide test kit (Beyotime Institute of Biotechnology, Shanghai, China). After adding 100 μL of the test solution to 50 μL of supernatant, the mixture was incubated for 20 min at room temperature. Absorbance at 560 nm was measured, and the H₂O₂ concentration was calculated using a standard curve.

### 2.8. Antioxidant Enzyme Activities Assay

The antioxidant enzyme activities were quantified as described by Yao et al. [23] with minor modifications. Homogenise the post-treated bacterial pellets in PBS (0.01 M, pH 7.2) and crush them using an ultrasonic disruptor (300 W, interval 5 s, 10 min), followed by centrifugation at 8000× *g* for 15 min at 4 °C. The supernatant was tested for Glutathione (GSH), Catalase (CAT), and Superoxide dismutase (SOD) activity using detection kits (A006-1-1, A007-1-1, A001-1-1, Jiancheng Bioengineering Institute, Nanjing, China). The GSH Assay Kit: the assay quantifies GSH by exploiting its sulfhydryl reactivity. GSH reacts with 5,5′-dithiobis to generate yellow 5-thio-2-nitrobenzoic acid, with absorbance measured at 420 nm.

The CAT Assay Kit: Catalase activity is determined by rapidly quenching H_2_O_2_ decomposition with ammonium molybdate, which simultaneously forms a yellow-coloured complex with residual H₂O₂, with absorbance measured at 405 nm.

The SOD Assay Kit: Superoxide anion radicals generated via the xanthine/xanthine oxidase reaction system oxidise hydroxylamine to form nitrite, which develops a purple-red colour with a chromogenic agent for spectrophotometric measurement. In the presence of SOD, the enzyme specifically inhibits Superoxide anion radicals generation, resulting in reduced nitrite formation and a lower absorbance in test tubes compared to controls, with absorbance measured at 550 nm.

### 2.9. Analysis of Key Enzyme Activities and Adenosine Triphosphate (ATP) Content in the Tricarboxylic Acid Cycle

The key enzyme activities and ATP content were quantified by He et al. [24]. The post-treated bacterial pellets were resuspended in PBS (0.01 M, pH 7.2). Subsequently, ultrasonic processing (300 W, interval 5 s, 10 min) was selected to release intracellular enzymes, including Succinate dehydrogenase (SDH), Malate dehydrogenase (MDH), and ATP content, which were determined using detection kits (A022-1-1, A021-2-1, A095-1-1, Jiancheng Bioengineering Institute, Nanjing, China), following the instructions.

The SDH Assay Kit: SDH catalyses the substrate reaction in which FAD is a cofactor, and FAD is reduced to FADH, which is coupled to the reduction of 2,6-DPIP, and the determination of the rate of reduction of 2,6-DPIP can be used to deduce the activity of SDH, with absorbance measured at 600 nm.

The MDH Assay Kit: The redox reaction catalysed by MDH is accompanied by a decrease in absorbance at 340 nm, and the activity of MDH was calculated by measuring the change in absorbance.

The ATP Assay Kit: Creatine kinase catalyses the reaction between adenosine triphosphate and creatine to produce phosphocreatine. The amount of phosphorus produced is measured by phosphomolybdic acid colorimetry, and the amount of ATP is calculated, with absorbance measured at 636 nm.

### 2.10. Gas Chromatography–Mass Spectrometry (GC-MS)

A 5.0 g sample was placed in a 20 mL headspace vial, then in a thermostatic water bath. The ageing syringe needle was inserted into the sealed vial, and the sample was extracted at 60 °C for 1 h. After extraction, the vial was immediately inserted into the injection port of the GC-MS system for thermal desorption for 3 min. The extraction head was conditioned at 250 °C for 5 min before each extraction to minimise memory effects.

GC conditions: A TG-5MS column (30 m × 0.25 mm × 0.25 μm) was used; the carrier gas was high-purity helium (99.999%); the flow rate was 1.2 mL/min; a splitless injection was employed; the injector temperature was 250 °C. The temperature programme started at 40 °C, was held for 2 min, and then ramped at 6 °C/min to 280 °C, where it was held for 4 min. MS conditions: Electron impact (EI) ionisation; transfer line temperature: 280 °C; ion source temperature: 300°C; electron energy: 70 eV; scan range (*m*/*z*): 33–500 amu, with full scan mode.

Qualitative analysis was performed using Xcalibur 4.2 software to compare the obtained spectra with the NIST 17 mass spectrum library. Quantitative analysis was carried out using the peak area normalisation method in the NIST library data processing system to calculate the percentage composition of each volatile compound in the crayfish sample.

### 2.11. Data Analysis

Each experiment was performed in triplicate, and data are presented as mean ± standard deviation. Statistical analysis was conducted using SPSS (version 19.0, SPSS Inc., Chicago, IL, USA) with one-way ANOVA, where *p* < 0.05 was considered significant. Graphs were generated using Origin 2021 and Excel 2016.

## 3. Results and Discussion

### 3.1. The Optimal Preparation Conditions for US-PAW

**Figure 1:**
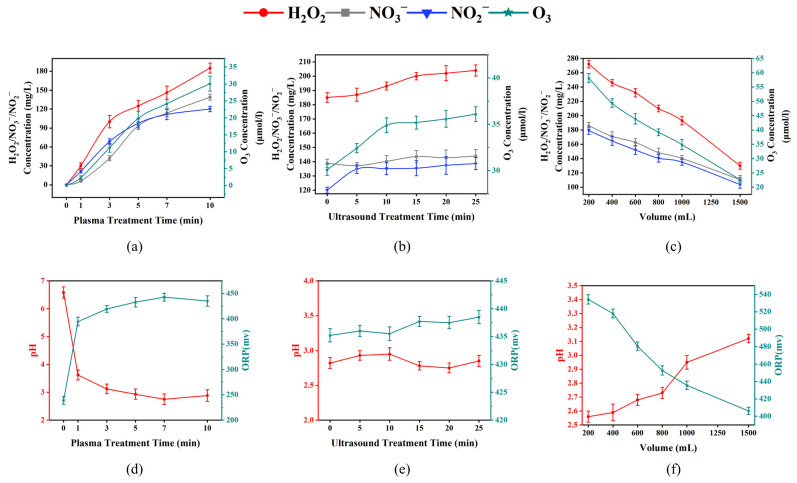
The effects of plasma treatment time, ultrasound treatment time, and liquid volume on the physicochemical properties of US-PAW. H_2_O_2_/NO_2_^−^/NO_3_^−^/O_3_ concentrations of different plasma treatment times (**a**), different ultrasound treatment times (**b**), different liquid volumes (**c**); pH and ORP values of different plasma treatment times (**d**), different ultrasound treatment times (**e**), different liquid volumes (**f**).

The physicochemical indices of US-PAW for the PAW treatment time (0, 1, 3, 5, 7 and 10 min), US treatment time (0, 5, 10, 15, 20, and 25 min), and water volume (200, 400, 600, 800, 1000, and 1500 mL) are presented in Figure 1.

As shown in Figure 1a,d, with an increasing plasma treatment time (0–10 min), H_2_O_2_, O_3_, NO_2_^−^, NO_3_^−^, and ORP rapidly increased to 185 mg/L, 30.08 μmol/L, 120 mg/L, 138.85 mg/L, and 435.2 mV, respectively, while pH decreased sharply to 2.88. These changes gradually levelled off over time. In comparison, the concentrations of H_2_O_2_, O_3_, NO_2_^−^, NO_3_^−^, and ORP in PAW increased slowly during 0 to 25 min of US treatment. Moreover, no significant difference in pH was observed in PAW during 0 to 25 min of US treatment (Figure 1b,e). In contrast, the content of H_2_O_2_, O_3_, NO_2_^−^, NO_3_^−^, and ORP in PAW dramatically decreased with volume. During PAW preparation, pH is positively correlated with volume (Figure 1c,f).

The same results have also been obtained in many other studies. Among them, the significant pH drop is likely due to the generation of NO_2_^−^ and NO_3_^−^ during plasma discharge [25]. ORP can reflect the total oxidation level of a solution [23]. Therefore, the increase in ORP is likely due to the rise in H_2_O_2_ and O_3_ concentrations. In addition, the degree of the ORP increase and pH decrease was correlated with the degree of water ionisation [26]. There was a slight increase in the ORP, H_2_O_2_, O_3_, NO_2_^−^, and NO_3_^−^ of the PAW with the rise in US treatment time, probably due to the US-induced acoustic dissociation of water and the production of free radicals (e.g., -H and -OH), which reactivated the residual free radicals in the PAW [10]. Acidic conditions and high ROS are key to antibacterial efficacy, with their interactions synergistically enhancing the antimicrobial activity of US-PAW [9].

To achieve higher antimicrobial activity (increased ORP, H_2_O_2_, O_3_, NO_2_^−^, NO_3_^−^, and reduced pH), the optimised conditions for US-PAW generation were established as 10 min of plasma treatment, 10 min of US treatment, and a 1000 mL processing volume, based on the physicochemical properties observed in this study.

### 3.2. The Inactivation of A. veronii by US-PAW

**Figure 2:**
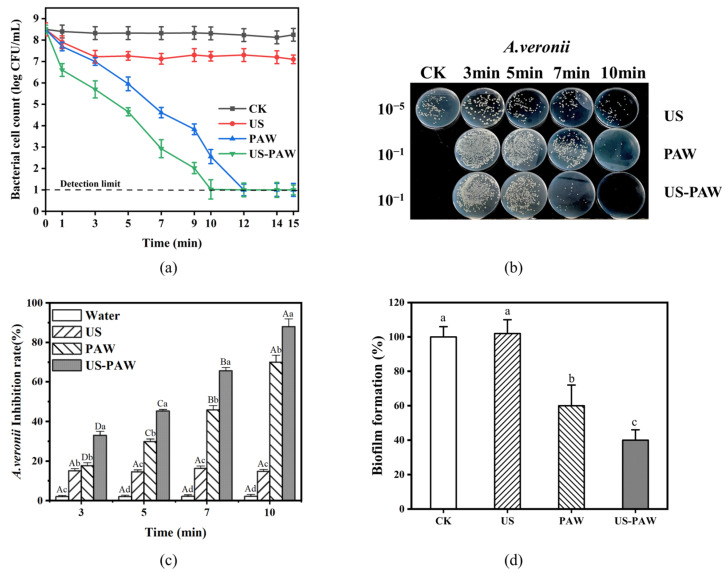
The time–kill curves (**a**) and inhibition rate (**b**) of US and PAW alone or in combination against *A. veronii*; (**c**) the inhibition rate; (**d**) the inhibition of biofilm reformation effects of US and PAW alone or in combination against *A. veronii* at 7 min. Different lowercase letters indicate significant (*p* < 0.05) differences between treatment groups, while different uppercase letters indicate significant (*p* < 0.05) differences between times.

The inhibition efficiency of US, PAW, and US-PAW against *A. veronii* in 0–10 min was measured. Figure 2a,c shows that when the treatment time was increased from 0 to 10 min, the inactivating effects of *A. veronii* in the water and US groups were kept at a low level, and the changes were insignificant (*p* < 0.05). In contrast, the inhibition rate of *A. veronii* by PAW and US-PAW was increased from 17.67 to 69.93% and 32.99 to 87.94% as the treatment time increased, respectively, and these were both significantly higher (*p* < 0.05) than that of the water and US groups at the corresponding time.

Previous studies have reported similar findings on the antimicrobial effects of treatments against *Aeromonas*. For example, nonthermal plasma treatment for 10–30 min achieved 99.98% bactericidal activity against *Aeromonas hydrophila* [27]. Additionally, PAW generated by Dielectric Barrier Discharge can effectively reduce *Aeromonas spp.* in fish over the long term without adversely affecting fish growth [28].

In this study, it is worth noting that the gap (45.77–69.93%) in inhibition rate between PAW and US-PAW is most apparent at 7 min. Therefore, a treatment time of 7 min was selected to verify the synergistic effect of US-PAW and perform the following experiments.

### 3.3. Effect of US-PAW on the Regrowth Capacity of A. veronii Biofilm

PAW has been shown to rapidly inactivate biofilms in the short term and inhibit biofilm regeneration [29,30]. However, evaluating the biofilm regeneration ability of surviving bacteria after US-PAW treatment reflects its long-term antimicrobial effect on *A. veronii* biofilms, which is crucial for assessing US-PAW’s potential to inhibit food contamination and secondary wound infections driven by *A. veronii* biofilms. The combination of US and PAW effectively inhibited the regrowth of *A. veronii* biofilms (Figure 2d), and the effect was significantly better than that of US and PAW used alone (*p* < 0.05). Interestingly, although US did not decrease the biofilm formation of *A. veronii*, US-PAW almost completely suppressed biofilm formation, better than PAW alone. These results are consistent with those of Charoux et al. [31], who found that combining airborne acoustic ultrasound with PAW enhanced its ability to inhibit biofilm formation. Furthermore, the findings align with the inactivation efficiency of US-PAW against *A. veronii* (Figure 2c), further confirming that US-PAW not only suppresses *A. veronii* growth but also inhibits biofilm formation.

### 3.4. Scanning Electron Microscopy Observations

**Figure 3:**
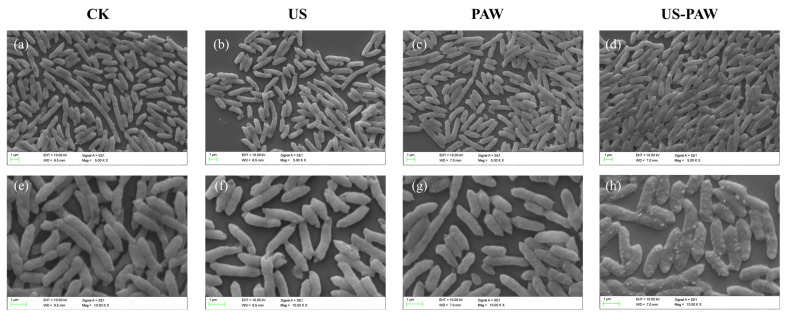
The microstructure of *A. veronii* under SEM (EVO-LS10) after US and PAW alone or in combination treatments for 7 min. (**a**,**e**) the SEM of *A. veronii* untreated (5000× and 10,000×). (**b**,**f**) the SEM of *A. veronii* treated by US (5000× and 10,000×). (**c**,**g**) the SEM of *A. veronii* treated by PAW (5000× and 10,000×). (**d**,**h**) the SEM of *A. veronii* treated by US-PAW (5000× and 10,000×).

As shown in Figure 3, the micromorphology of *A. veronii* was observed via SEM. For the CK group, the cells of *A. veronii* appeared to be rodlike, with smooth and intact edges and surfaces. For the US group, some *A. veronii* surface edges showed slight damage. After PAW and US-PAW treatments, the cells of *A. veronii* exhibited apparent changes in morphology, such as fold breakages and blurred edges. Studies have shown that PAW treatment induces morphological changes in *Aeromonas* observed through SEM images [32]. This is similar to this finding, where a transition from a smooth surface to distortion, shrinkage, and rupture is evident. US-PAW treatment caused more pronounced morphological changes in *A. veronii*, with white pores appearing on the surface (Figure 3h), likely due to US enhancing PAW’s cell membrane disruption and subsequent release of cellular contents. The effect of US-PAW on the cell membrane was further examined to confirm this.

### 3.5. Effects of US-PAW on the Cell Membrane

#### 3.5.1. Cell Membrane Integrity

**Figure 4:**
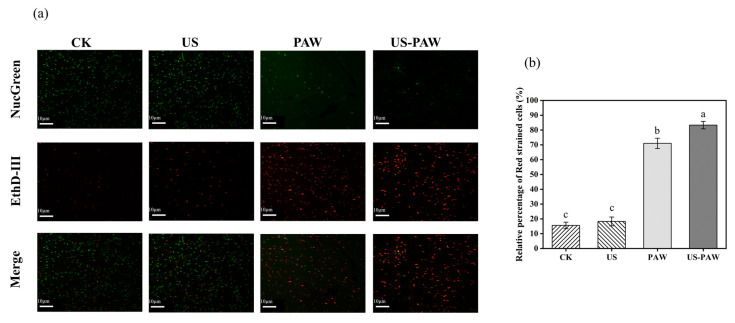
The cell membrane integrity of *A. veronii* under CLSM by different treatments. The EthD-III/NucGreen double-staining images (**a**) and relative percentage of red-stained cells (**b**). Different lowercase letters indicate significant (*p* < 0.05) differences between treatment groups.

A NucGreen/EthD-III double-staining assay was performed to verify the synergistic effect of US and PAW on membrane integrity using CLSM (Figure 4a). NucGreen is a green nucleic acid dye that stains live bacteria with intact cell membranes; EthD-III is a red nucleic acid dye that only stains dead bacteria with damaged cell membranes [33]. With the CK and US treatment, most of the *A. veronii* cells were stained by NucGreen, and only a few were stained by EthD-III (15.58 and 18.27%). By contrast, after treatment with PAW, *A. veronii* partially excited green fluorescence, and the staining rate of red cells was 71.01%. When US and PAW were combined, *A. veronii* elicited stronger red fluorescence (83.33%) than PAW (Figure 4b), indicating a synergistic effect on cell membrane damage, resulting in rapid cell death.

#### 3.5.2. Electrical Conductivity of *A. veronii* Suspensions

**Figure 5:**
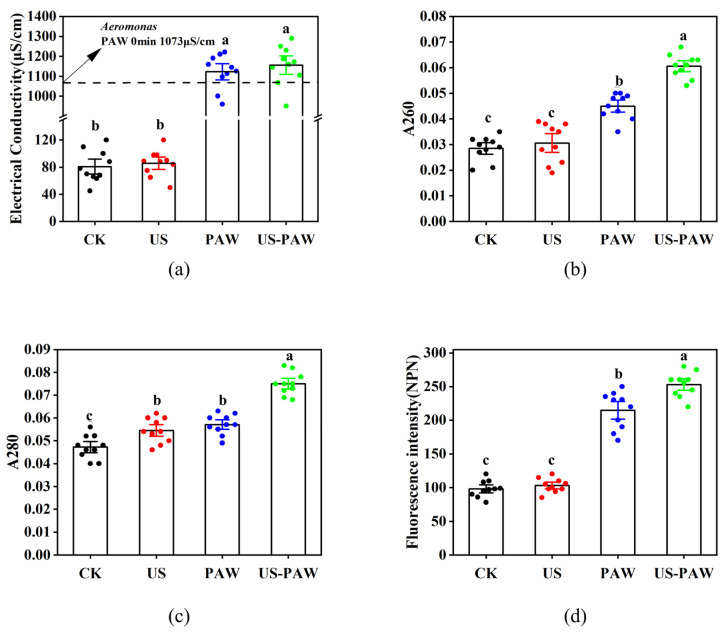
Changes in the membrane permeability of *A. veronii* treated with US and PAW alone or in combination for 7 min. (**a**) Electrical conductivity; (**b**) extracellular nucleic acids; (**c**) extracellular proteins; (**d**) outer membrane permeability. Different lowercase letters indicate significant (*p* < 0.05) differences between treatment groups. The scatter points on the columns represent each specific piece of data in the group.

Next, the electrical conductivity of *A. veronii* suspensions after US, PAW, and US-PAW treatments was examined to understand the synergistic effect on cell membrane permeability [34]. The results in Figure 5a indicate that the electrical conductivity value inside the bacterial cells increased after 7 min of treatments. After US treatment, the electrical conductivity of *A. veronii* suspensions increased to 85.72 μS/cm, and there was no significant difference compared with the CK group. However, after PAW and US-PAW treatment, the electrical conductivity of *A. veronii* suspensions dramatically increased to 1122 μS/cm and 1155 μS/cm, respectively. In this study, when US and PAW were combined, the electrical conductivity of *A. veronii* suspensions was not significantly different compared with the PAW alone, indicating that they did not have an apparent synergistic effect on cell membrane permeability damage from the electrical conductivity aspect.

#### 3.5.3. Protein and Nucleic Acid Leakage

The absorbances at 260 and 280 nm are conventionally utilised for quantifying DNA and protein concentrations, respectively, and can further serve as indicators of intracellular DNA and protein release, as well as compromised cell membrane permeability. As shown in Figure 5b,c, the A260 and A280 values of *A. veronii* increased significantly after US-PAW treatment, reaching 0.0606 and 0.075, respectively (*p* < 0.05). As a result, the US aggravates the damage of PAW to cell membrane permeability, leading to more leakage of intracellular proteins and nucleic acids. Still, the level of leakage was not significant.

#### 3.5.4. Permeability of the Outer Membrane

Gram-negative bacteria are surrounded by both an inner and an outer membrane. The outer membrane serves as a key protective barrier, preventing harmful environments or toxic compounds from affecting the cell. As such, it is a critical target in antibacterial research for Gram-negative bacteria [35]. NPN, a hydrophobic fluorescence probe, emits weak fluorescence in aqueous solutions. However, it cannot efficiently penetrate the bacterial outer membrane under normal conditions, making it difficult to access the internal environment. NPN only permeates and interacts with internal phospholipids to emit strong fluorescence when the outer membrane is damaged, forming pores or defects [36].

The NPN fluorescence probe method was used to assess the impact of US-PAW on the outer membrane permeability of *A. veronii*. As shown in Figure 5d, compared to the CK and US-only groups, the NPN fluorescence intensity of *A. veronii* was significantly higher in the PAW and US-PAW groups (*p* < 0.05). After PAW treatment, the NPN fluorescence intensity of *A. veronii* increased from 98.1 to 214.5; after US-PAW treatment, it increased from 98.1 to 253.1. These results indicated that US treatment alone did not significantly enhance NPN fluorescence intensity, while US-PAW significantly increased it compared to PAW alone. In agreement with this study, previous research has shown that PAW enhanced the outer membrane permeability in *Pseudomonas deceptionensis CM2* [37]. Overall, US-PAW exhibited a significant synergistic effect on disrupting the outer membrane permeability of *A. veronii*, providing evidence for its mechanism of increasing membrane permeability and inducing cell death.

### 3.6. Generation of ROS and H_2_O_2_

**Figure 6:**
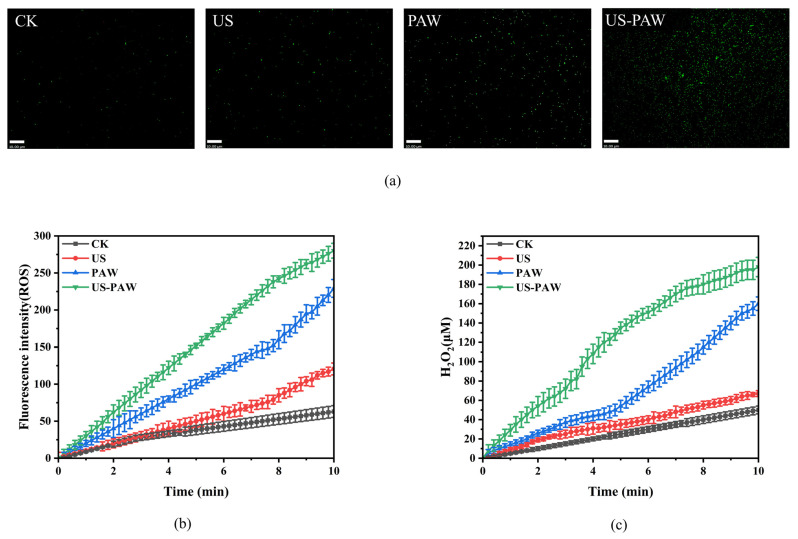
Images of intracellular ROS in *A. veronii* after different treatments by CLSM (**a**). Changes in intracellular ROS (**b**) and H_2_O_2_ (**c**) in *A. veronii* induced by US and PAW alone or in combination for 10 min.

To further evaluate the inhibitory mechanism of US-PAW against *A. veronii*, the intracellular ROS and H_2_O_2_ content were determined (Figure 6b,c). US treatment slightly increased the intracellular ROS levels in *A. veronii*, likely due to the cavitation effect generating ROS in water, which then enters the cells through mechanical forces [38]. There were apparent increases in the intracellular ROS and H_2_O_2_ levels in *A. veronii* after PAW treatment. Interestingly, US-PAW treatment significantly elevated intracellular ROS and H_2_O_2_ levels in *A. veronii* to 280 a.u./198 μM, surpassing those of the US (120.6 a.u./67 μM) and PAW (229.1 a.u./160 μM) treatments. In addition, CLSM was used to observe *A. veronii* after US, PAW, and US-PAW treatment. As expected, the fluorescence changes were consistent with the measured fluorescence intensities (Figure 6a). These data suggested that US promoted the ability of PAW to induce ROS and H_2_O_2_ accumulation, leading to cell damage and cell death.

### 3.7. Effects of US-PAW on the Intracellular Antioxidant Enzyme System

**Figure 7:**
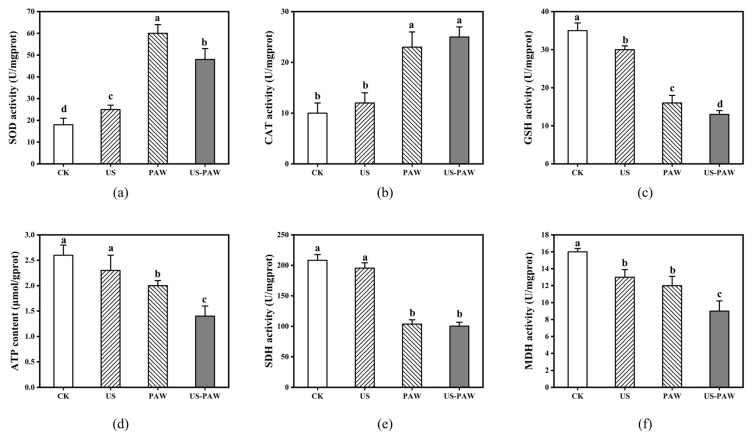
The effects of US and PAW alone or in combination for 7 min on the intracellular antioxidant enzyme and energy metabolic system. Antioxidant enzyme system: (**a**) SOD activity; (**b**) CAT activity; and (**c**) GSH activity. Energy metabolic system: (**d**) ATP content; (**e**) SDH activity; and (**f**) MDH activity. Different lowercase letters indicate significant (*p* < 0.05) differences between treatment groups.

The antioxidant system is a key defence mechanism that regulates antioxidant levels, including enzymes like SOD and CAT and non-enzymatic antioxidants such as GSH, to maintain cellular redox balance and protect against external stress [39]. As shown in Figure 7a–c, the SOD activity in the CK and US groups was 18.24 and 25.44 U/mg prot, respectively. PAW treatment significantly increased SOD activity to 60.18 U/mg prot, while US-PAW treatment reduced it to 48.53 U/mg prot. This may be due to SOD being the first line of defence against oxidative damage [40]. No significant difference in CAT activity was observed between the PAW and US-PAW groups. After PAW treatment, CAT activity increased from 10.24 to 23.51 U/mg prot, and it increased to 25.3 U/mg prot after US-PAW treatment. US and PAW treatments reduced the GSH content from 35.32 to 30.15 and 16.21 mg/mg prot, respectively. In contrast, the GSH content after US-PAW treatment was 13.09 mg/mg prot, significantly lower than the individual treatments (*p* < 0.05).

Notably, PAW treatment alone significantly increased the activity of SOD and CAT, indicating a defensive state and enhanced antioxidant capacity. However, when intracellular ROS accumulate and exceed a certain threshold, toxic ROS remain unneutralised and damage the antioxidant enzymes, disrupting cellular homeostasis [41]. US-PAW treatment significantly reduced SOD activity and GSH levels, indicating that the reactive species might exceed a certain threshold, impairing antioxidant enzyme function and disrupting the antioxidant system.

### 3.8. Effects of US-PAW on the Energy Metabolic System

The tricarboxylic acid cycle (TCA) is a primary energy source and a central metabolic pathway in bacteria. Key enzymes in the TCA cycle play essential roles and are important targets for antimicrobial agents. SDH, located on the mitochondrial inner membrane, supplies electrons to the respiratory chain and is a key enzyme in oxidative phosphorylation and electron transport. MDH facilitates the interconversion of malate and oxaloacetate. ATP content and SDH and MDH activities reflect the organism’s energy metabolism status.

As shown in Figure 7d–f, ATP content in the US group was similar to that in the CK group, while PAW and US-PAW treatments reduced ATP levels to 2.06 μmol/g prot and 1.42 μmol/g prot, respectively. This study aligns with Liao et al., who reported that plasma treatment-induced oxidative stress consumes more cellular energy [42]. Additionally, SDH connects the electron transport chain and the TCA cycle. Previous studies have shown that plasma treatment inhibited 35% of SDH activity in *Pseudomonas aureofaciens* [43]. In this study, the CK group had an SDH activity of 208.30 U/mg prot. After US, PAW, and US-PAW treatments, SDH activity decreased to 195.41 U/mg prot, 103.67 U/mg prot, and 100.42 U/mg prot, respectively. This decline in SDH activity indicates disruption of the electron transport chain, further reducing energy synthesis. Finally, MDH activity in the CK group was 16.20 U/mg prot. US and PAW treatments reduced it to 13.14 U/mg prot and 12.48 U/mg prot, with no significant difference. US-PAW treatment further decreased MDH activity to 9.26 U/mg prot. Research has indicated that MDH is crucial for metabolism and the exchange of cellular reductants, and its activity decline reflects reduced cellular metabolic levels [44]. Overall, the disruption of the respiratory chain and decreased cellular metabolism reduced TCA cycle efficiency, affecting the energy supply, with the ATP reduction confirming this result. Thus, US-PAW treatment might further limit the TCA cycle rate, significantly impacting *A. veronii* respiration and energy metabolism.

### 3.9. Effects of US-PAW on Crayfish During Storage at 4 °C

#### 3.9.1. Antibacterial Effects on Crayfish

**Figure 8:**
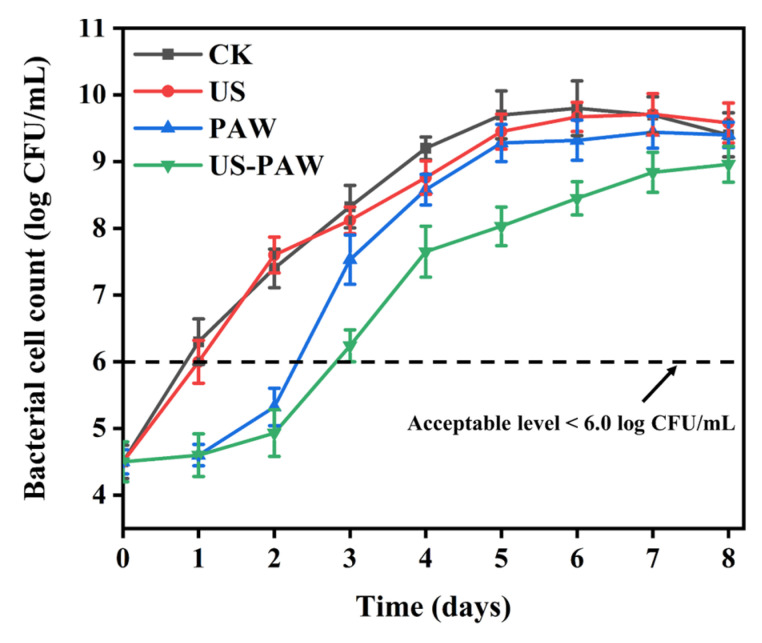
Effect of US and PAW on the survival counts of *A. veronii* in crayfish during storage at 4 °C alone or in combination.

The above results indicated that US-PAW could efficiently inactivate the predominant spoilage bacterium *A. veronii* in vitro. Nonetheless, the antibacterial effect and duration of US-PAW disinfection on *A. veronii* on crayfish remain unknown. As shown in Figure 8, the bacterial cell count of *A. veronii* in all treatment groups increased over time during storage at 4 °C. Compared with the CK group, treatments with PAW and US-PAW inhibited bacterial growth during storage. Specifically, treatment with US-PAW was the most effective against *A. veronii*. Generally, the critical value of bacterial cell counts for the spoilage of meat samples stored at 4 °C is (8.11 ± 0.29) log CFU/g [45]. However, a bacterial cell count below 6.00 log CFU/g is considered the acceptable level for microbial quality in aquatic products [46]. The populations of the CK group reached 6.00 log CFU/g on day 1 of storage, while the US-PAW group only surpassed this on day 3, extending the storage time by 2 days compared to the CK group. After 4 days of storage, the populations of *A. veronii* treated with US-PAW were 7.65 ± 0.38 log CFU/g, obviously lower (*p* <0.05) than the CK, US, and PAW groups (9.2 ± 0.17 log CFU/g, 8.76 ± 0.25 log CFU/g, and 8.58 ± 0.23 log CFU/g, respectively). No significant difference in *A. veronii* populations was observed after 4 days of storage in the CK, US, and PAW groups. Total volatile basic nitrogen (TVBN) as an important indicator of spoilage in aquatic products is closely related to bacterial counts. This finding is consistent with previous studies, which showed that US-PAW treatments typically lead to more significant reductions in the natural microbiota of crayfish and TVBN values during storage [11]. Our results indicated that US-PAW is effective in inactivating *A. veronii* in vitro, more effectively inhibiting its growth on crayfish and delaying spoilage 2 days during storage.

#### 3.9.2. Effect of US-PAW on Volatile Flavour Compounds of Crayfish

Volatile flavour compounds are typically produced through enzymatic reactions, lipid auto-oxidation, microbial activity, environmental contamination, and thermal reactions [47]. GC-MS identified fifty volatile flavour compounds from crayfish stored at 4 °C for 6 days in both the CK and US-PAW groups. The compounds included sulphur-containing compounds, ketones, N, O-containing compounds, acids, hydrocarbons, alcohols, esters, phenolics, and others (Table 1).

Sulphur compounds, key components of aroma in many foods, may originate from sulphur-containing amino acids (free amino acids, peptides, and protein amino acids) [48]. Carbon disulphide, hydrogen sulphide, and dimethyl disulphide indicate potential bacterial spoilage in packaged seafood [49,50]. Compared to the CK group, S-containing compounds in the US-PAW group decreased significantly from 72.16% to 47.82%. This reduction may be attributed to inhibiting spoilage bacteria that decompose sulphur-containing amino acids caused by ROS, reactive nitrogen species (RNS) from PAW, and US cavitation and mechanical effects.

Ketones in seafood are typically produced from the oxidation or degradation of unsaturated fatty acids and amino acids [51]. Alcohols, known as secondary lipid oxidation products, primarily originate from the degradation of polyunsaturated fatty acids [52]. The increase in compounds like ketones and alcohol in the US-PAW group might have resulted from PAW accelerating the oxidation of unsaturated fatty acids. Due to their high odour thresholds, ketones and alcohols have a limited impact on the overall flavour.

Hydrocarbons are typically derived from alkyl groups by lipid autoxidation and often have aromatic or sweet notes [53]. Xylene, detected in several seafood products like fish miso and grass carp, is thought to result from the metabolism of aromatic amino acids such as tryptophan, phenylalanine, and tyrosine or from environmental contamination [54]. Overall, the hydrocarbon content showed a minimal change between the CK and US-PAW groups and had little effect on the overall flavour.

Esters are typically produced through microbial activity or the esterification of carboxylic acids and alcohols during lipid metabolism [55]. This study’s ester with the highest relative content was 2,2,4-trimethyl-1,3-pentanediol diisobutyrate, followed by ethyl and acetate esters. These compounds are considered quality indicators for fish products and can be used to assess the level of spoilage [56]. The main N, O-containing compound identified was Oxime-, methoxy-phenyl-_. Although research on Oxime-, methoxy-phenyl-_ is limited, it has been detected in some aquatic and meat products, such as grass carp and Chinese mitten crab [51]. The increase in N, O-containing compounds in the experimental group may be attributed to the effects of ROS and RNS in the PAW.

In conclusion, GC-MS analysis suggested that US-PAW treatment preserves the flavour characteristics of crayfish during storage.

## 4. Conclusions

This study demonstrated that optimised conditions for US-PAW generation were established as 10 min of plasma treatment, 10 min of US treatment, and a 1000 mL processing volume. In addition, the combination of US and PAW effectively inhibited biofilm reformation and had a bactericidal effect against *A. veronii* at 7 min. The proposed synergistic antibacterial mechanism of US-PAW involved bacterial distortion, deformation, and the formation of cavities and holes. This leads to the leakage of intracellular substances, disruption of cell membranes, and increased membrane permeability, and it ultimately inhibited bacterial growth following the combined treatment. Additionally, the therapy increased intracellular H_2_O_2_ and ROS levels and disrupted cellular homeostasis. The decrease in SOD and GSH activities further exacerbated oxidative stress. At the same time, reduced ATP, MDH, and SDH activities impair the cell’s ability to generate energy, making it unable to resist external stress, leading to bacterial death. US-PAW treatment extended the shelf life of crayfish (infected with *A. veronii*) by 2 days and reduced sulphur-containing volatiles by 24.64% during a 6-day storage at 4 °C, suggesting its potential as a novel preservation strategy for crayfish.

## Figures and Tables

**Table 1:** Volatile flavour compounds of crayfish at day 6 of storage at 4 °C after US-PAW treatment.

ID.	Volatile Compounds	Relative Amount (%)	
		CK	US-PAW
1	Sulphur-containing compounds	72.16	47.82
2	Ketones	1.61	17.08
3	N, O-containing compounds	1.11	8.49
4	Acids	0.1	3.43
5	Hydrocarbons	4.11	5.99
6	Alcohols	0	4.03
7	Esters	8.48	8.74
8	Phenolic	0.11	0.86
9	Others	12.32	3.56

## Data Availability

The original contributions presented in this study are included in the article. Further inquiries can be directed to the corresponding author.

## Abbreviations

ATP	Adenosine triphosphate
*A.veronii*	*Aeromonas veronii*
CAT	Catalase
CK	Untreated
CLSM	Confocal laser scanning microscope
GC-MS	Gas Chromatography–Mass Spectrometry
GSH	Glutathione
H_2_O_2_	Hydrogen peroxide
MDH	Malate dehydrogenase
NPN	N-Phenyl-1-naphthylamine
ORP	Oxidation–reduction potential
PAW	Plasma-activated water
RNS	Reactive nitrogen species
ROS	Reactive oxygen species
SDH	Succinate dehydrogenase
SEM	Scanning electron microscopy
SOD	Superoxide dismutase
TCA	Tricarboxylic acid cycle
TVBN	Total volatile basic nitrogen
US	Ultrasound
US-PAW	Ultrasound combined with plasma-activated water
